# Bromide impairs the circadian clock and glycolytic homeostasis via disruption of autophagy in rat H9C2 cardiomyocytes

**DOI:** 10.1186/s12860-020-00289-8

**Published:** 2020-06-19

**Authors:** Yicheng Jiang, Yang Gu, Hai Xu, Xiaoyi Tian, Xuefeng Zhang, Xiaojin Xu, Wenting Yan, Xiwen Zhang

**Affiliations:** The Affiliated Huai’an No.1 People’s Hospital of Nanjing Medical University, No. 1 Huanghe West Road, Huaiyin District, Huai’an, 223300 Jiangsu China

**Keywords:** Bromide, H9C2, Circadian clock, Glycolysis, Autophagy

## Abstract

**Background:**

Trace elements function as essential cofactors that are involved in various biochemical processes in mammals. Autophagy is vital for nutrient supplement, which is an important *Zeitegber* for the circadian homeostasis in heart. Here, we considered the possibility that autophagy, as well as the cardiomyocyte clock and glycolysis are interlinked. Detrimental effects were observed when cardiac system is exposed to bromine containing drugs. This study investigated the effects and mechanisms of bromide on the circadian clock and glycolytic metabolism of H9C2 cardiomyocytes.

**Results:**

In the present study, bromide does not affect cell viability and apoptosis of H9C2 cardiomyocytes. Bromide dampens the clock and glycolytic (*Hk2* and *Pkm2*) gene expression rhythmicity in a dose-dependent manner. Additionally, bromide inhibits autophagic process in H9C2 cardiomyocytes. In contrast, rapamycin (an autophagy inducer) dramatically restores the inhibitory effect of NaBr on the mRNA expression levels of clock genes (*Bmal1*, *Cry1* and *Rorα*) and glycolytic genes (*Hk2* and *Pkm2*).

**Conclusions:**

Our results reveal that bromide represses the clock and glycolytic gene expression patterns, partially through inhibition of autophagy.

## Background

Sufficient trace elements play important roles in maintaining our body healthy. Recently, it has been well established that the deficiency of metal ions, such as iron, zinc and copper, leads to various diseases including heart failure, diabetes and bone marrow hematopoiesis [1–3]. Of note, bromine is a unique trace element that possessing negative charge in the biosphere. In the past twentieth century, the environmental doses of bromide increased due to the salt-mining wastes and the degradation products of fumigants, causing an inevitably exposure of bromide for the general population [4]. Hence, as a residue in food, the toxicological and physiological evaluations of bromide are raring necessary.

Bromide is widely used in the drug development. For example, triple bromide elixir functions as an adjunctive antiepileptic drug to treat the children whose seizure disorders were intractable to other antiepileptic therapy [5]. Tiotropium bromide (Spiriva®) is a long-term anticholinergic bronchodilator that maintains bronchodilation for at least one day [6]. More importantly, the concentrations of bromide are negatively correlated with the lipids including TG, TC, and HDL-C in the human and rat plasma [6]. Coincidence with these findings, bromide exhibits beneficial effects on the FFA-induced lipid dysregulation in mouse hepatocytes_,_ increasing its possibility in the treatment of metabolic disorders such as hepatic steatosis [7]. All these studies implicate that bromide could be developed as a promising drug in the treatment of various diseases. However, the neuromuscular blocking drugs (NMBD), pancuronium bromide shows a short-lasting cardiovascular stimulation after a high-dose injection, indicating the potential detrimental effects of bromide on the cardiac system [8]. Given that bromide penetrates the cell membrane through the chloride channel which is a vital ion channel in the cardiovascular system [9, 10], it is of great interest to track the pharmacological evidences of bromide on cardiac system and its underlying mechanisms.

For decades, the circadian clock has been demonstrated to maintain the cardiac homeostasis, such as heart rate and blood pressure [11]. Clinical investigations indicate that circadian disruption in shift workers is associated with increased cardiovascular morbidity and mortality blood pressure variation shift workers [12]. These findings are confirmed by animal studies that cardiomyocyte-specific Bmal1 knockout and Clock mutant mice suffer from age-onset cardiomyopathy, early mortality and sinus bradycardia [13]. On the other hand, to meet energy demands, cardiomyocytes dynamically reprogram fuel and energize their metabolic capacity in response to environmental and physiological cues [14]. Importantly, the substrate availability varies in a 24-h (hour) day, which directly influences the energy supplement. Collectively, these findings demonstrate a tight link between the circadian clock and homeostasis of the cardiac system. However, in general, the molecular process which integrates the cardiac circadian clock and metabolism in response to various external stimulus, such as bromide, remains unknown. Here, we aimed to investigate the effects of bromide on the H9C2 cardiomyocyte survival and its circadian physiological homeostasis with the hope of elucidating the mechanism of its cardiovascular medicinal potential.

## Results

### Bromide does not affect survival and apoptosis of H9C2 cardiomyocytes

**Fig. 1:**
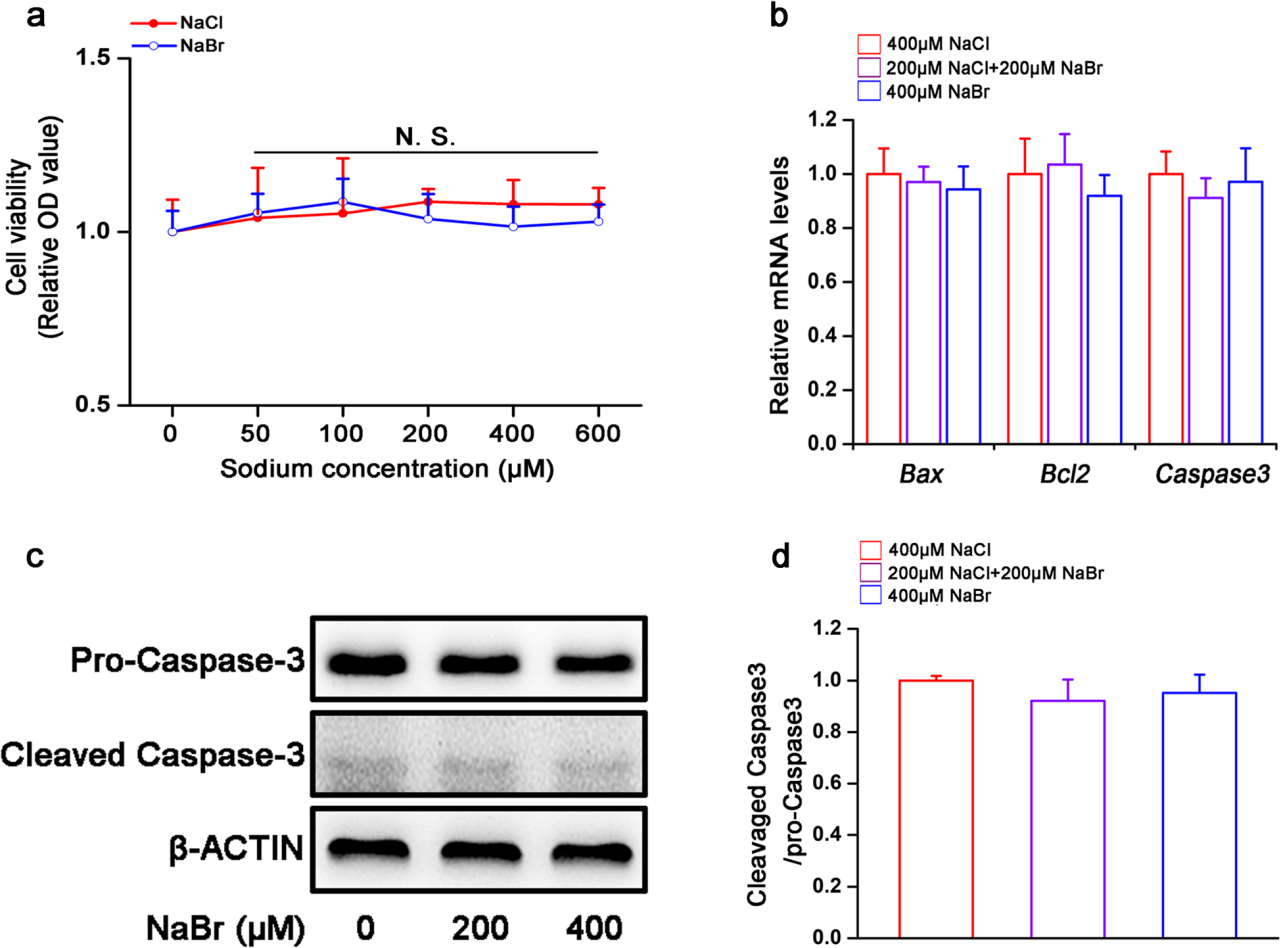
Bromide does not affect survival and apoptosis of H9C2 cardiomyocytes. H9C2 cardiomyocytes were treated with NaBr at indicated doses for 24 h. **a** Cell viability was assessed by CCK-8 assay. **b** RT-qPCR analysis of the mRNA expression levels of *Bax*, *Bcl2* and *Caspase-3*. **c** Western blot analysis of protein expression levels of Caspase-3. **d** Densitometric determinations of (c). *n* = 3. All the data were represented as the mean ± SD. N.S. means no significance

To investigate the effects of bromide on the cardiomyocytes, we firstly assessed the toxicity of NaBr. As shown in Fig. 1a, CCK-8 analysis demonstrated that NaBr was not toxic to H9C2 cardiomyocytes when the concentration was up to 600 μM. Besides, similar tendency was observed in isolated rat neonatal primary cardiomyocytes when treated with the same doses of NaBr (Fig. S1). Hence, the doses range from 50 μM to 400 μM were regarded as safe and were chosen for the subsequent experiments. In addition, bromide did not affect mRNA expression levels of apoptosis-associated factors *Bcl-2*, *Bax* and *Caspase-3* (Fig. 1b). Consistently, the active form level of Caspase-3 (the cleaved type) was not altered by NaBr incubation (Fig. 1c and d). These results implied that cell apoptosis was not regulated in response to bromide stimulation.

### Bromide dampens clock gene expression in H9C2 cardiomyocytes

**Fig. 2:**
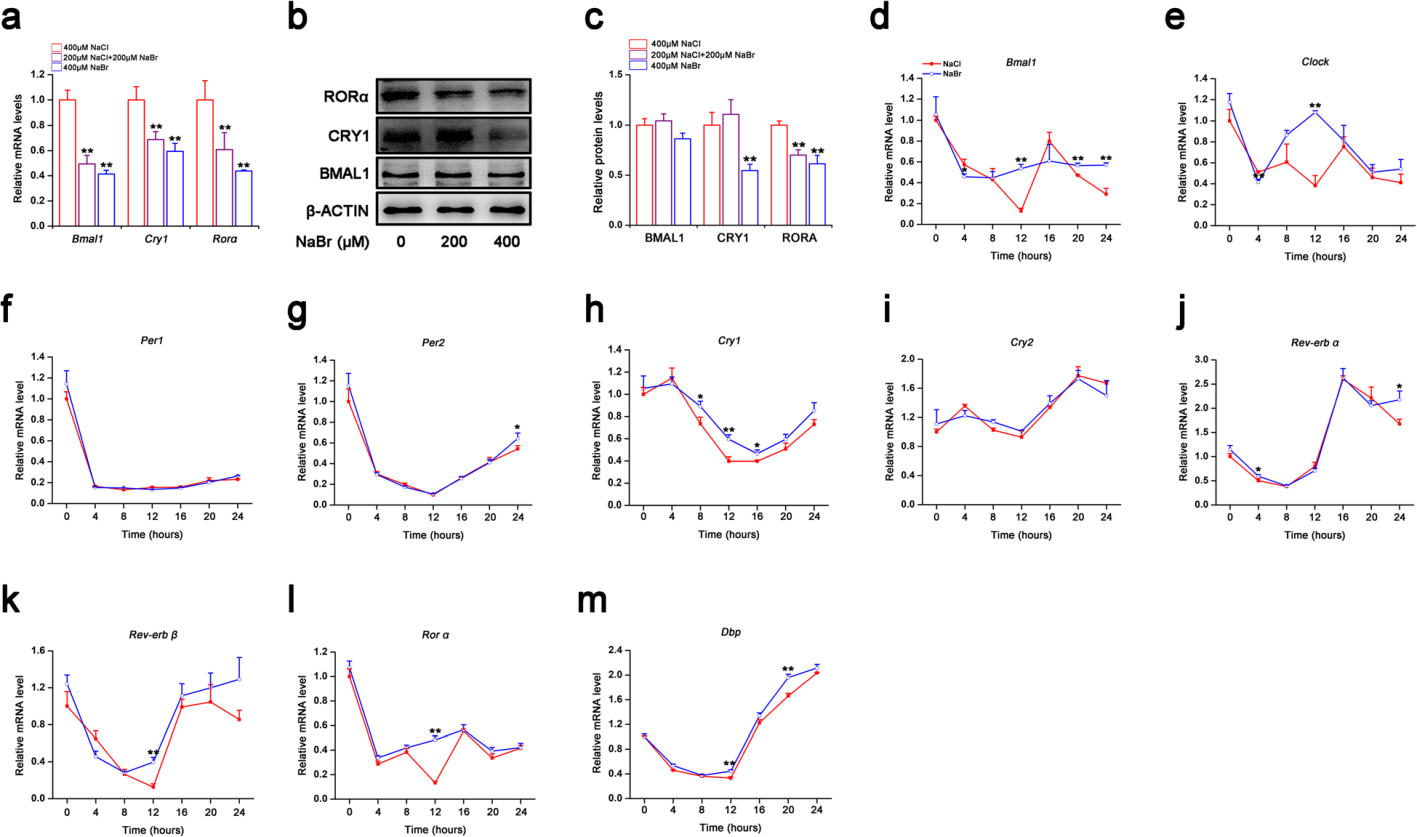
Bromide dampens clock gene expression in H9C2 cardiomyocytes. H9C2 cardiomyocytes were incubated with NaBr at indicated doses for 24 h. **a** RT-qPCR analysis of the mRNA expression levels of *Bmal1*, *Cry1* and *Rorα*. **b** Western blot analyses of protein expression levels of BMAL1, CRY1 and RORα. **c** Densitometric analyses of (**b**). (d-m) RT-qPCR analysis of the mRNA expression levels of clock genes in serum-shocked H9C2 cardiomyocytes treated with or without 400 μM NaBr. *p < 0.05 and **p < 0.01 vs. NaCl group. *n* = 3. All the data were represented as the mean ± SD

As shown in Fig. 2a and b, treatment of NaBr at the doses of 200 μM and 400 μM robustly inhibited mRNA levels of key clock genes *Bmal1*, *Cry1* and *Rorα* in a dose-dependent manner. In particular, 400 μM NaBr inhibited mRNA levels of *Bmal1* by 41.5%, *Cry1* by 59.5% and *Rorα* by 43.8% respectively. The protein expression of these genes showed similar trends in response to NaBr (Fig. 2a-c). Also, we detected other clock genes expression upon NaBr treatment in Fig. S2. Of note, serum shock has been demonstrated to induce rhythmic clock gene expression in various cells. Here, in our system, serum shock also resulted in a robust oscillation of clock genes including *Bmal1*, *Cry1* and *Rorα.* etc. (Fig. 2d, h and l). However, the *Per1* did not exhibit an obvious circadian oscillation in H9C2 cardiomyocytes, which is in consistent with previous findings (Fig. 2f). Notably, NaBr treatment did not alter the phase of oscillation patterns of clock genes, but dampened the amplitudes at most checked time-points, except for *Clock*, whose amplitudes was intensified by NaBr incubation (Fig. 2d-m and Table S1). All these findings suggested the detrimental role of bromide in dampening the circadian clock in H9C2 cardiomyocytes.

### Bromide inhibits glycolytic gene expression in H9C2 cardiomyocytes

**Fig. 3:**
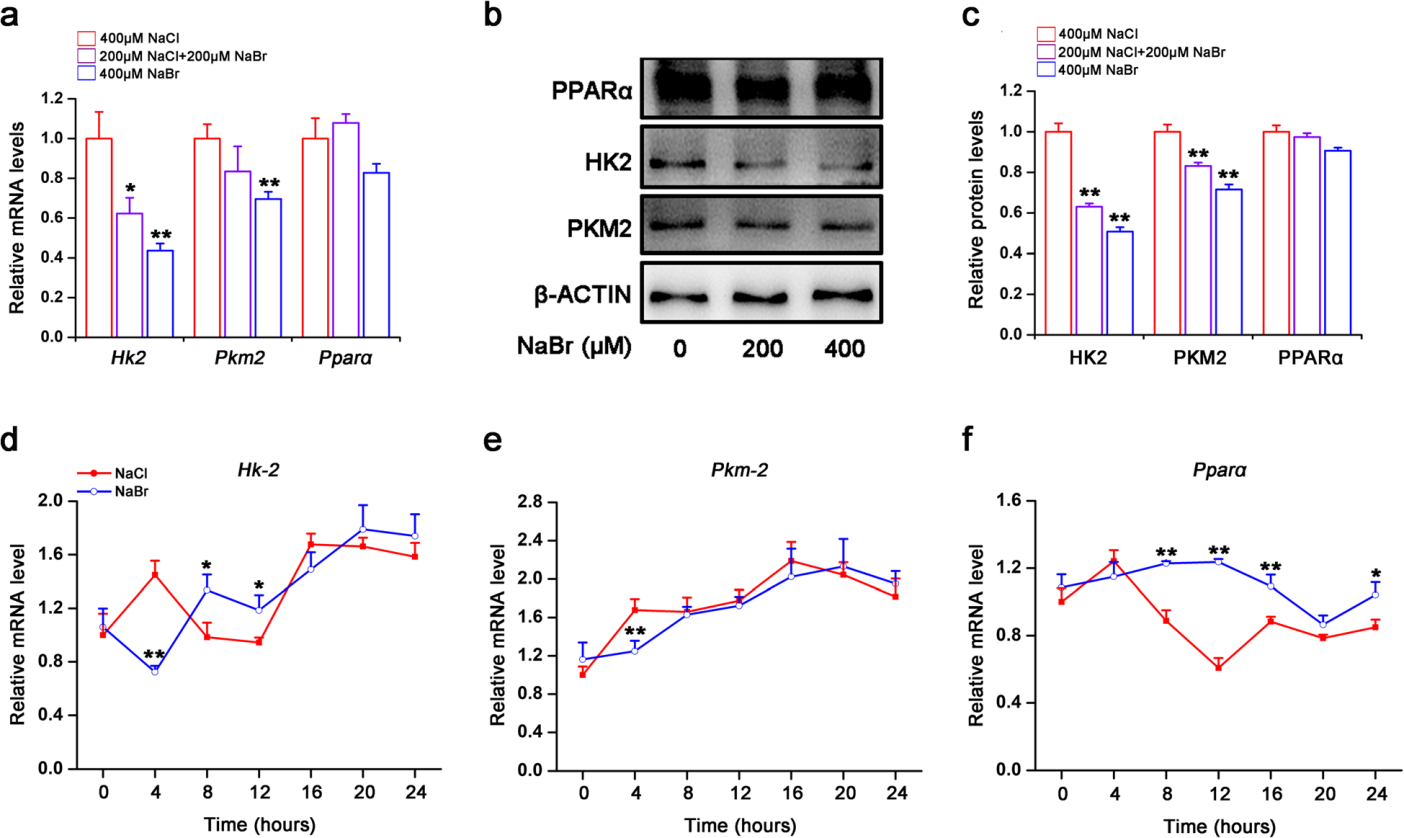
Bromide inhibits glycolytic gene expression in H9C2 cardiomyocytes. H9C2 cardiomyocytes were treated similar as in Fig. 2a. **a** RT-qPCR analysis of the mRNA expression levels of *Hk2*, *Pkm2* and *Pparα*. **b** Western blot analysis of protein expression levels of HK2, PKM2 and PPARα. **c** Densitometric determinations of (**b**). (d-f) RT-qPCR analysis of the mRNA expression levels of *Hk2*, *Pkm2* and *Pparα* in serum-shocked H9C2 cardiomyocytes treated with or without 400 μM NaBr. *p < 0.05 and **p < 0.01 vs. NaCl group. *n* = 3. All the data were represented as the mean ± SD

Given that circadian disruption in cardiomyocytes is closely correlated with the transition of fuel utilization from lipid oxidation to glycolysis [15], it is of great interest to identify the impact of bromide on the glycolysis. Unexpectedly, NaBr did not increase, however, dose-dependently decreased mRNA expression levels of *Hk2* and *Pkm2*, which are hallmarks of glycolysis (Fig. 3a). Similar results were obtained in the protein levels of these genes (Fig. 3b and c). In contrast, PPARα, an important transcriptional factor that activates fatty acid β-oxidation in heart, was not altered upon NaBr incubation (Fig. 3a-c). Furthermore, serum shock also induced significant oscillation of *Hk2, Pkm2* and *Pparα* mRNAs. However, bromide dampened the amplitudes of *Pkm2* and *Pparα,* while leaving *Hk2* unchanged in both its phase and amplitude, compared to NaCl-treated group (Fig. 3d-f and Table S2).

### Bromide inhibits autophagy in H9C2 cardiomyocytes

**Fig. 4:**
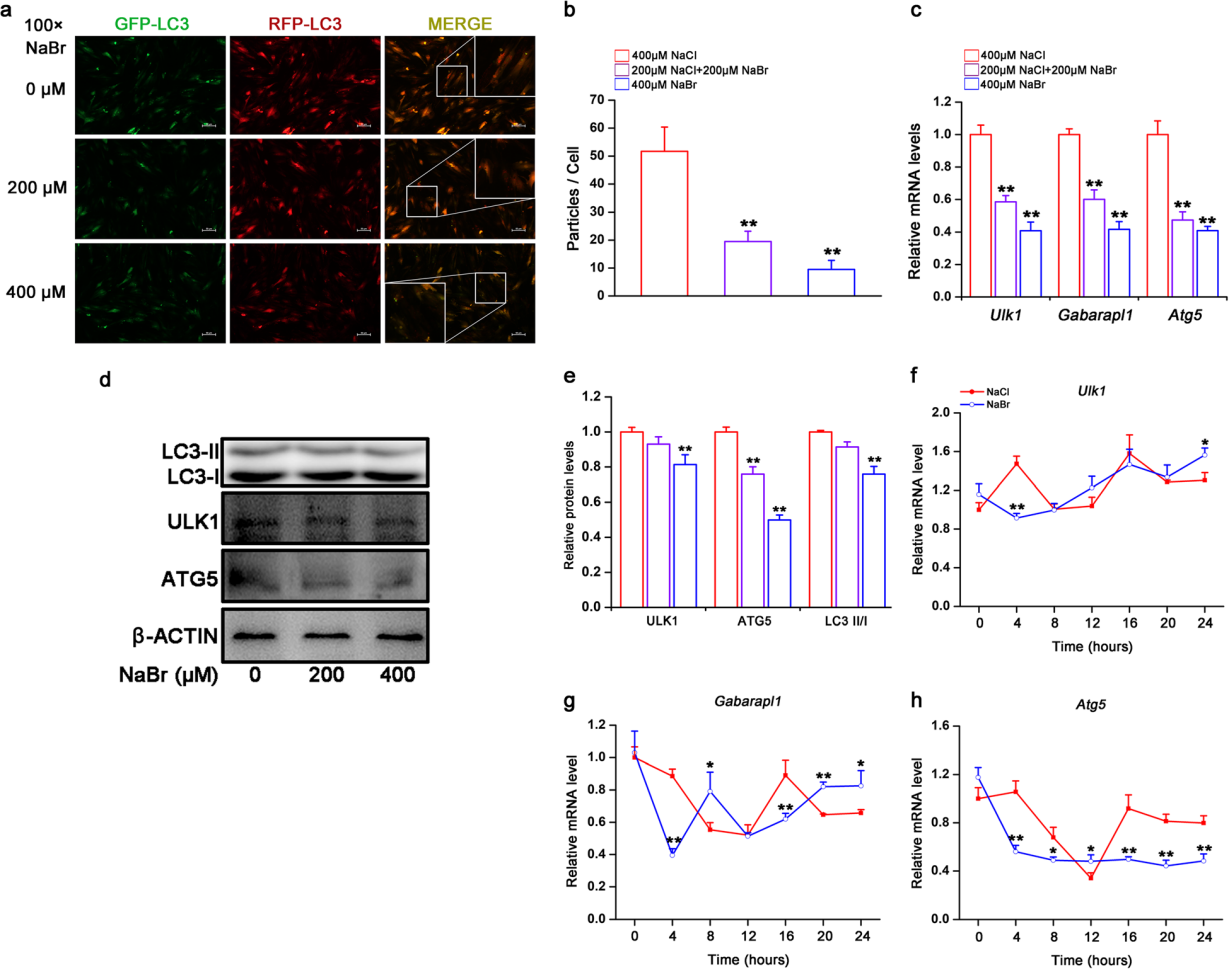
Bromide inhibits autophagy in H9C2 cardiomyocytes. **a** H9C2 cardiomyocytes were infected with the adenovirus expressing GFP-RFP-LC3 for 24 h, and followed by NaBr stimulation for another 24 h. Magnification: 100×. H9C2 cardiomyocytes were treated similar as in Fig. 2a. **b** Analysis of the images from the experiment shown in Fig. 4a. to determine the average number of particles per cell. **c** RT-qPCR analysis of the mRNA expression levels of *Ulk1, Gabarapl1* and *Atg5*. **d**, **e** Western blot and densitometric analyses of protein expression levels of LC3, ULK1 and ATG5. **f**-**h** RT-qPCR analysis of the mRNA expression levels of *Ulk1*, *Gabarapl1* and *Atg5* in serum-shocked H9C2 cardiomyocytes treated with or without 400 μM NaBr. *p < 0.05 and **p < 0.01 vs. NaCl group. n = 3. All the data were represented as the mean ± SD

Autophagy is a cellular process that delivers cytosolic components to lysosomes for degradation in response to metabolic stress, such as starvation, to provide a source of nutrients and metabolic fuel [16]. As shown in Fig. 4a and b, NaBr dramatically reduced the formation of autophagic puncta evidenced by using adenovirus expressing GFP-RFP-LC3. Coincidence with these findings, NaBr significantly reduced the LC3 II/LC3 I ratio and mRNA expression levels of *Ulk1*, *Gabarapl1* and *Atg5*, which are key factors in regulating the autophagosome formation (Fig. 4c). Consistently, the protein expression levels of ULK1 and ATG5 were inhibited by NaBr in a dose-dependent manner (Fig. 4d and e). In addition, serum shock successfully induced rhythmic oscillation of *Ulk1*, *Gabarapl1* and *Atg5* genes expression in H9C2 cells. While the amplitudes of *Ulk1* and *Atg5* were dampened, bromide modestly altered the *Gabarapl1* expression oscillation pattern (Fig. 4f-h and Table S3).

### Autophagy mediates the inhibitory effect of bromide on the circadian clock and glycolytic gene expression in H9C2 cardiomyocytes

**Fig. 5:**
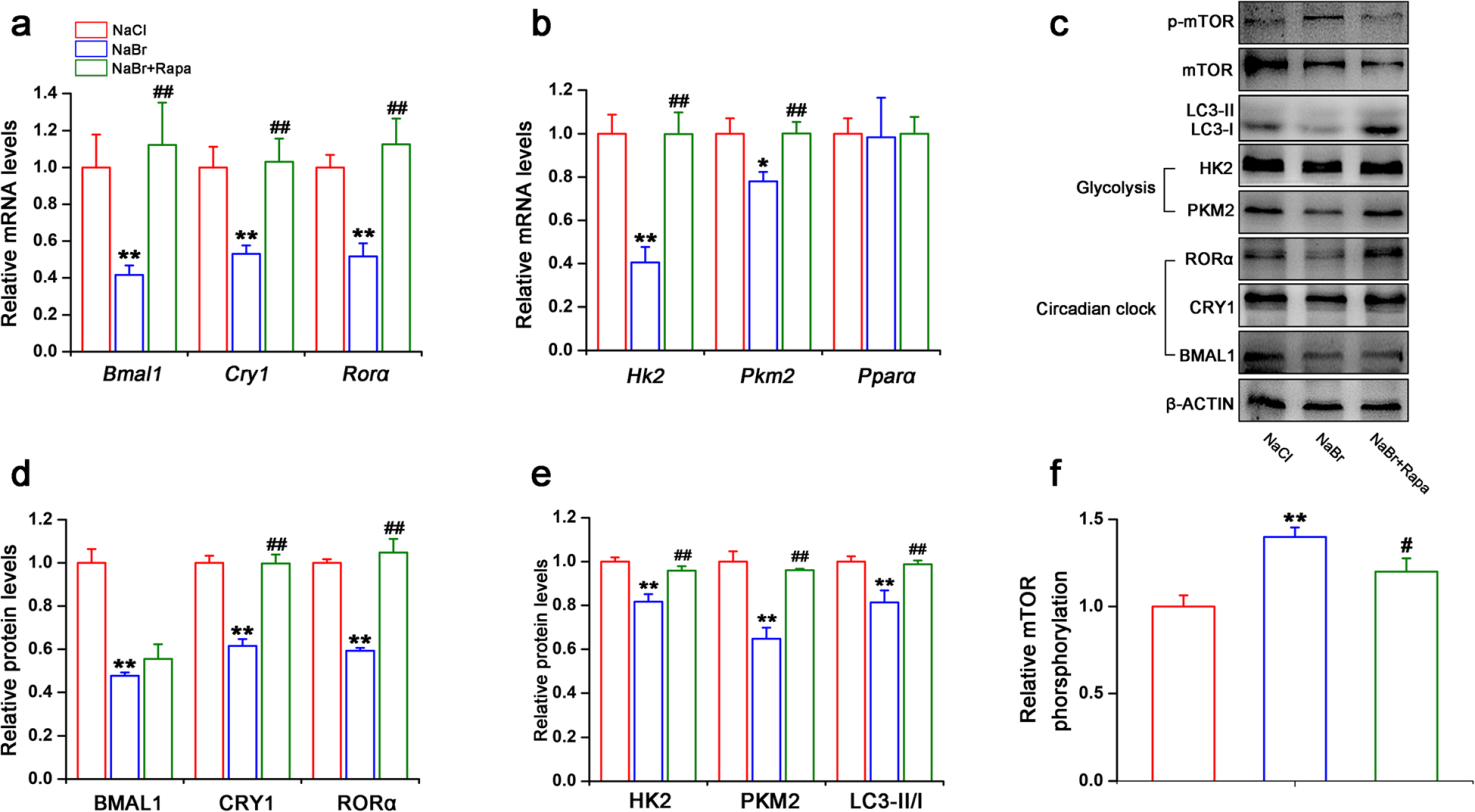
Autophagy mediates the inhibitory effect of bromide on the circadian clock and glycolytic gene expression in H9C2 cardiomyocytes. H9C2 cardiomyocytes were treated with 400 μM NaBr in combination with or without 100 nM rapamycin for 24 h. **a** RT-qPCR analysis of the mRNA expression levels of *Bmal1*, *Cry1* and *Rorα*. **b** RT-qPCR analysis of mRNA expression levels of *Hk2*, *Pkm2* and *Pparα*. **c** Western blot analysis of protein expression levels of LC3, BMAL1, CRY1, RORα, PKM2, HK2, p-mTOR and mTOR. **d** Densitometric determinations of BMAL1, CRY1, RORα, **e** HK2 and PKM2. **f** Densitometric determination analyses of phosphorylation levels of mTOR. *p < 0.05 and **p < 0.01 vs. NaCl group, ^#^*p* < 0.05 and ^##^*p* < 0.01 vs. NaBr group. n = 3. All the data were represented as the mean ± SD

**Fig. 6:**
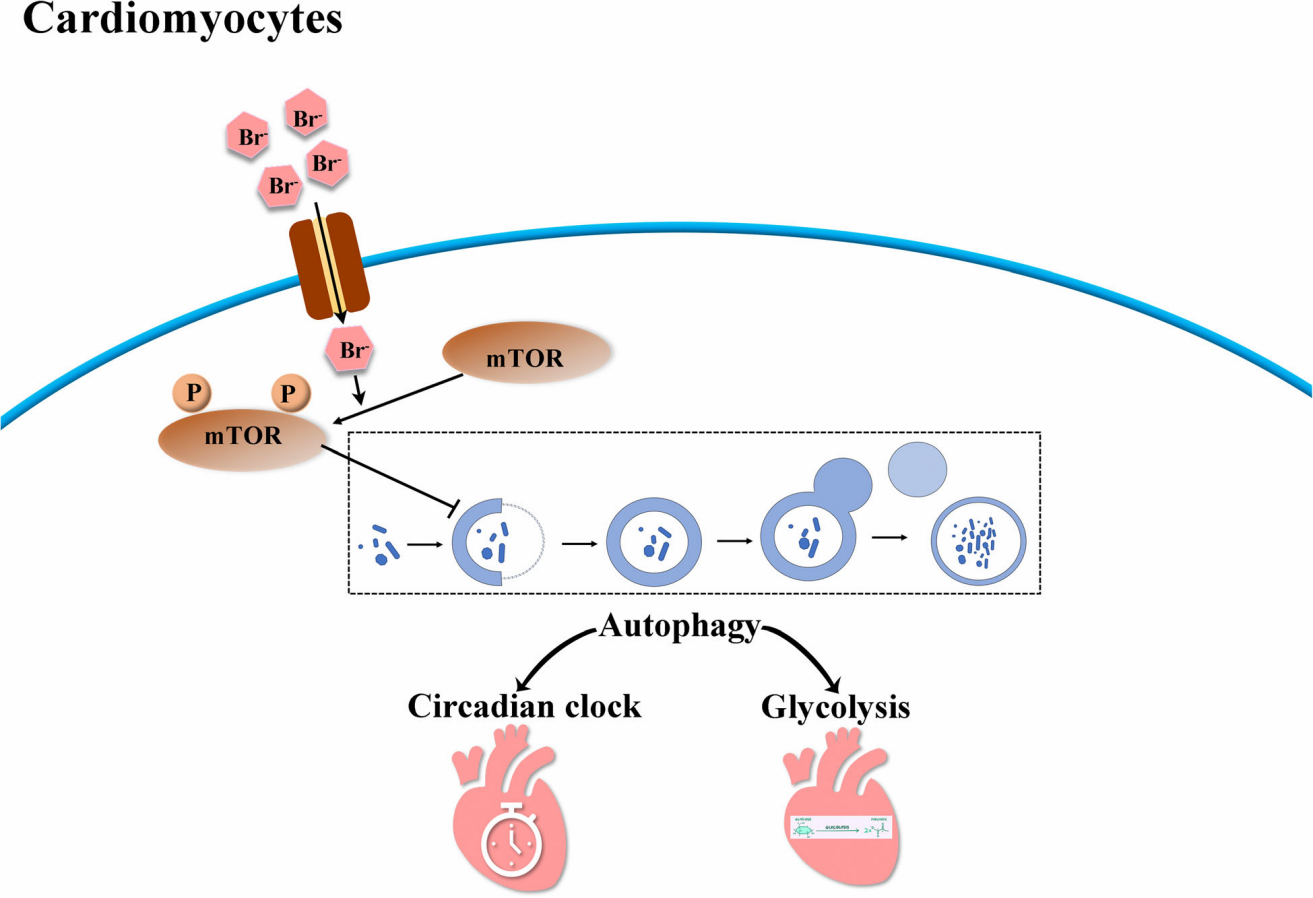
The functional model illustrating the mechanism by which bromide dampens circadian and glycolytic gene expression and rhythmicity through inhibition of the autophagy in H9C2 cardiomyocytes, highlighting the mediating role of mTOR in the bromide signal relay

To investigate the role of autophagy in the regulation of metabolism and autophagy in H9C2 cardiomyocytes, we incubated cells with 100 nM rapamycin (inhibitor of mTOR activity, as an autophagy inducer). As shown in Fig. 5a and b, rapamycin restored the inhibitory effect of NaBr on the mRNA expression levels of clock genes (*Bmal1*, *Cry1* and *Rorα*) and glycolytic genes (*Hk2* and *Pkm2*). Also, protein levels of these genes showed similar tendency (Fig. 5c-e). Additionally, the phosphorylation of mTOR protein was slightly inducd by NaBr treatment (increased to ~ 1.4 folds), which was then retarded by rapamycin incubation (Fig. 5c and f), indicating that bromide may inhibit autophagy partially through activating mTOR pathway, and further dampening clock and glycolytic gene expression and their rhythmicity (Fig. 6). In addition, another autophagy inducer, QX77, also partially reversed the inhibitory effects of NaBr on the mRNA and protein expression levels of clock genes (*Bmal1*, *Cry1* and *Rorα*) and glycolytic genes (*Hk2* and *Pkm2*) (Fig. S3). Given that the autophagy is vital for the maintaining homeostasis in the physiological state, we treated H9C2 cardiomyocytes with 1 μM H_2_O_2_ and 400 μM NaBr. As shown in Fig. S4, NaBr-treated H9C2 cardiomyocytes are susceptible to H_2_O_2_ stimuli, indicating that the bromide senses the cardiomyocytes to external toxic signals.

## Discussion

As an essential trace element, bromide maintains the redox homeostasis at a serum concentration of 42 ~ 61 μM in healthy individuals [17, 18]. Clinically, high dose bromide (~ 2.1 mM) is used to treat the epilepsy. In 1990s, bromide was thought to be safe with a LD50 at 7000 mg/kg in rats after acute gavage [19]. However, potential toxicity was observed in the endocrine and reproductive system. Animal experiments revealed that 90-day consecutive gavage of high-dose bromide (14,900 mg/kg) increased the weights of thyroid adrenal and prostate and induced pathological changes in pituitarium, thyroid, testicle and ovary [19]. More importantly, detrimental effects were observed in cardiac system. For example, bromide-containing drugs pancuronium which is a neuromuscular-blocking drug, induces temporary cardiovascular stimulation, accompanied with rapid up-regulations in heart rate, average arterial blood pressure and cardiac output in rats [8]. Rats feed with diet containing 0.8% brominate vegetable oil (BVO) show enlarged heart and dissolved cardiomyocytes, further inducing the degenerative changes of the myocardium [20]. In our study, we found that bromide did not alter the survival and apoptosis of H9C2 cardiomyocytes, however, affected the clock and metabolic homeostasis. All these findings indicated the cardiac system are sensitivity to bromide stimulation. Hence, bromide may have potential harmful effect on the cardiac system.

Given the potential toxic effects of bromide on the cardiovascular system, our study was aimed to clarify the impact of bromide on the cardiomyocytes in vitro. In our study, we found that bromide did not alter the cell viability and apoptosis, whereas decreased the autophagy and glycolysis. More importantly, treatment of bromide increased susceptibility to the H_2_O_2_-induced toxicity of H9C2 cardiomyocytes. On the other hand, chronotherapy and chronopharmacology, which functioned to minimize the drug toxicity and maximize drug efficacy and tolerance, are based on the circadian system. Therefore, we investigated the effect of bromide on the cardiomyocytes’ clock to provide experimental and theological basis for the potential effective timing for the bromide-containing drugs in the future study.

Autophagy is a crucial evolutionarily conserved biological process responsible for eliminating long half-life proteins, damaged organelles and pathogens [21]. Autophagy deficiency has been demonstrated in the pathogenesis of various diseases, such as cancer [22], diabetes [23], and heart failure [24]. Thus, autophagy is vital for cellular homeostasis and nutrient supplement. Given that the nutrient is a important *Zeitegber* for the circadian homeostasis in heart, here we considered the possibility that autophagy, as well as the cardiomyocyte clock and glycolysis are interlinked. In our study, rapamycin-induced autophagy increased the clock and glycolytic gene expression in response to bromide stimulation, indicating that autophagy indeed integrates the circadian clock and glycolysis in H9C2 cells. On the other hand, trace elements serve as a pivotal factor to regulate autophagy. For example, the aggravating effect of selenium deficiency on T-2 toxin-induced damage on primary cardiomyocyte results from a reduction of protective autophagy [25]. Therefore, bromide, as a unique trace element, may correlate with autophagic process in the heart. In our study, we found that bromide inhibited autophagic pathway through increasing the phosphorylation of mTOR protein. In contrast, activation of autophagy by rapamycin retarded bromide-induced impairment of the circadian clock and glycolysis in H9C2 cells, implicating the mediator roles of autophagy in bromide signals. At the molecular level, autophagy induces clock gene CRY1 protein degradation to regulate the liver clock and glucose metabolism [26]. However, rapamycin-induced autophagy transcriptionally increased the CRY1 expression in H9C2 cells, implying that autophagy regulates CRY1 expression at both posttranscriptional and transcriptional levels according to the tissue specificity. The effects of autophagy in mediating the bromide signals to the circadian clocks and glycolysis of H9C2 cardiomyocytes were further confirmed by another autophagic activator QX77.

## Conclusion

Our current discoveries demonstrated that the effects of bromide on the circadian and glycolytic gene oscillation in H9C2 cardiomyocytes, highlighting the mediating roles of autophagy/mTOR in the bromide signal relay. Our findings demonstrated the detrimental, but not toxic effects of high-dose bromide, and suggested that the potential side-effect of bromide-containing drugs on cardiac system. The cardiac safety of bromide should be considered in future drug development.

## Methods

### Cell culture

The rat H9C2 cardiomyocytes were cultured at 37 °C and 5% CO_2_ in Dulbecco’s Modified Eagle Medium (DMEM, high glucose, Gibco-Invitrogen, Carlsbad, USA), supplemented with 1% antibiotic-antimycotic (10,000 U/mL of penicillin, 10,000 mg/mL of streptomycin) and 10% fetal bovine serum (FBS, Gibco-Invitrogen), and were used between passages 15 to 25. To examine the autophagic status in response to bromide stimulation, we infected the cells with the adenovirus expressing GFP-RFP-LC3 for 24 h (hours), and treated with bromide or vehicle (equal molar sodium chloride) for another 24 h. The GFP-RFP-LC3-positive cells were examined by a Nikon fluorescence microscope (ECLIPSE, Ts2R-FL). Rat neonatal primary cardiomyocytes were isolated from the ventricles of Wistar rats aged 1–10 days (*n* = 10, purchased from Model Animal Research Center of Nanjing University, China) as previously described [27]. All animal procedures in this research conform to the Guide for the Care and Use of Laboratory Animals published by the US National Institutes of Health (NIH publication No. 85–23, revised 1996) and was approved by the Laboratory Animal Care & Use Committee at Nanjing Medical University (Permit number SYXK2018–0012). Briefly, newborn male Wistar rats (1–10 days old) were sacrificed by decapitation, hearts from rats were minced and dissociated with 0.25% trypsin and collagenase II. Dispersed cells were seeded at 10^5^ cells/well in 96-well plates with DMEM supplemented with 10% FBS and then cultured in a 5% CO_2_ incubator at 37 °C.

### CCK-8 toxicity assay

CCK-8 assay was performed to analyze potential toxic effects of sodium bromide (NaBr; Sigma-Aldrich, Germany) on H9C2 cardiomyocytes. Briefly, 1 × 10^4^ cells were seeded into each well of a 96-well plate and were cultured at 37 °C overnight. After synchronization with serum-free DMEM, cells were transferred into 100 μL serum-free DMEM containing either NaBr or equal amounts of sodium chloride (NaCl, positive control) at indicated concentrations (ranging from 10 μM to 600 μM) and incubated for another 24 h. Then, 10 μL CCK-8 reagent (Jiancheng, Nanjing, China) was added to each well and incubated at 37 °C for 4 h. Finally, a microplate reader was used to measure the absorbance at 450 nm.

### Serum shock

The media of confluent cultures was replaced with DMEM plus 50% horse serum. After 2 h shock, the cells were washed twice with PBS and incubated with serum-free DMEM containing 400 μM NaBr or NaCl. Cell samples were collected at 4-h intervals. Total RNA was extracted and processed for reverse transcription-quantitative polymerase chain reaction (RT-qPCR) analysis.

### RT-qPCR analysis

Total RNA from cells was isolated using Trizol reagent (Invitrogen, Carlsbad, California, USA), reverse transcribed with the PrimeScript RT reagent kit (Takara, Tokyo, Japan), and analyzed by real-time quantitative PCR using 2 × ChamQ Universal SYBR qPCR Master Mix (Vazyme, Nanjing, China) according to the manufacturer’s instructions. The Primers for rat GAPDH were included for normalization. A complete list of Primers was shown in Table S4 and synthesized by Generay Biotech Co., Ltd. (Shanghai, China).

### Western blotting analysis

For protein analysis, cells were lysed in RIPA buffer. The protein concentration was quantified with a BCA Protein Quantitation Assay Kit (Beyotime Biotech., Shanghai, China). Equal amounts of protein were loaded and separated by 10% SDS-PAGE and then transferred onto a PVDF membrane (Millipore Corp., Billerica, MA, USA). The membranes were incubated overnight with appropriate primary antibodies at 4 °C. Bound antibodies were then visualized using horseradish peroxidase-conjugated secondary antibodies. A quantitative analysis was performed by using ImageJ software (U.S. National Institutes of Health). For the antibody information, the antibodies against CRY1, HK2 and PKM2 were purchased from Proteintech (Chicago, IL, USA). Anti-RORα were obtained from Santa Cruz Biotechnology (CA, USA). The antibodies against LC3II/I, BMAL1, ULK1 and ATG5 were purchased from Bioworld Technology, Inc. (Nanjing, China). The antibody against β-ACTIN was derived from Servicbio Technology Co., Ltd. (Wuhan, China). The secondary antibodies were obtained from Santa Cruz Biotechnology (CA, USA). Uncropped images are shown in Fig. S5.

### Statistical analysis

Groups of data were presented as the means ± standard deviation (SD). Data were analyzed by using one-way ANOVA followed by Fisher’s LSD post hoc test. Calculations were performed by using Origin 8 software (version 8.6, OriginLab, Northampton, MA, USA). A value of *P* < 0.05 was considered statistically significant. Circadian variations, including amplitude andvphase shift, were calculated by fitting a cosine-wave equation [y = baseline + (Amplitude × Cos (2 × π × (x-phaseshift)/24)] on clock gene expression, with a fixed 24-h period (detailed data for the oscillation of clock genes were presented in Supplementary Table 2, 3 and 4). Time series data were analyzed using one-way or two-way ANOVA followed by Bonferroni’s post hoc test. A *p*-value of less than 0.05 was considered to be statistically significant. Unless otherwise indicated, the statistics was performed using Student’s t-test when only two groups were compared.

## Supplementary information

**Additional file 1: Figure S1.** Bromide does not affect survival and apoptosis of rat neonatal primary cardiomyocytes. Rat neonatal primary cardiomyocytes were treated with NaBr at indicated doses for 24 h. (a) Cell viability was assessed by CCK-8 assay.

**Additional file 2: Figure S2.** Bromide regulates clock gene expression in H9C2 cardiomyocytes. H9C2 cardiomyocytes were incubated with NaBr at indicated doses for 24 h. (a) RT-qPCR analysis of the mRNA expression levels of *Clock*, *Per1*, *Per2, Cry1, Rev-erbα, Rev-erbβ* and *Dbp*. *p < 0.05 and **p < 0.01 vs. NaCl group. *n* = 3. All the data were represented as the mean ± SD.

**Additional file 3: Figure S3.** QX77 partially reversed the inhibitory effect of NaBr on the mRNA and protein expression levels of clock genes and glycolytic genes. H9C2 cardiomyocytes were treated with 400 μM NaBr in combination with or without 10 μM QX77 for 24 h. (a) RT-qPCR analysis of the mRNA expression levels of *Bmal1*, *Cry1* and *Rorα*. (b) RT-qPCR analysis of mRNA expression levels of *Hk2*, *Pkm2* and *Pparα*. (c) Western blot analysis of protein expression levels of BMAL1, CRY1, RORα, PKM2 and HK2. (d) Densitometric determinations of BMAL1, CRY1, RORα, (e) HK2 and PKM2. *p < 0.05 and **p < 0.01 vs. NaCl group, ^#^*p* < 0.05 and ^##^*p* < 0.01 vs. NaBr group. *n* = 3. All the data were represented as the mean ± SD.

**Additional file 4: Figure S4.** H9C2 cardiomyocytes were susceptible to H_2_O_2_ stimuli after treatment of NaBr. H9C2 cardiomyocytes were treated with NaBr with or without 1 μM H_2_O_2_ for 24 h. Cell viability was assessed by CCK-8 assay. **p < 0.01 vs. NaCl group, ^##^*p* < 0.01 vs. NaCl plus H_2_O_2_ group. n = 3. All the data were represented as the mean ± SD.

**Additional file 5: Figure S5.** Uncropped images of the blots included in the Figures.

**Additional file 6: Table S1.** Calculations of Amplitude and Phase shift in Fig. 2. **Table S2.** Calculations of Amplitude and Phase shift in Fig. 3. **Table S3.** Calculations of Amplitude and Phase shift in Fig. 4. **Table S4.** The list of primer sequences for qPCR analysis.

## Data Availability

Data and materials used during the current study are available from the corresponding author on reasonable request.

## Abbreviations

CCK-8: Cell counting kit-8
NaBr: Sodium bromide
NaCl: Sodium chloride
GAPDH: Glyceraldehyde-3-phosphate dehydrogenase
CRY: Cryptochrome
HK2: Hexokinase 2
PKM2: Pyruvate kinase M 2
RORα: RAR-related orphan receptor α
BMAL1: Brain and muscle arnt-Like Protein 1
ULK1: Unc-51 like kinase 1
ATG5: Autophagy related 5
PPARα: Peroxisome proliferator activated receptor α
Garabapl1: GABA(A) receptor associated protein like 1
mTOR: Mechanistic target of rapamycin kinase
Clock: Circadian locomotor output cycles kaput
Per: Period
Rev-erb: Nuclear receptor subfamily 1, group D
Dbp: D site albumin promoter binding protein
