# Supplementary figures and images for: Bromide impairs the circadian clock and glycolytic homeostasis via disruption of autophagy in rat H9C2 cardiomyocytes
Source: BMC Mol Cell Biol. 2020 Jun 19;21:44. doi: 10.1186/s12860-020-00289-8 (PMC7304218; doi:10.1186/s12860-020-00289-8)

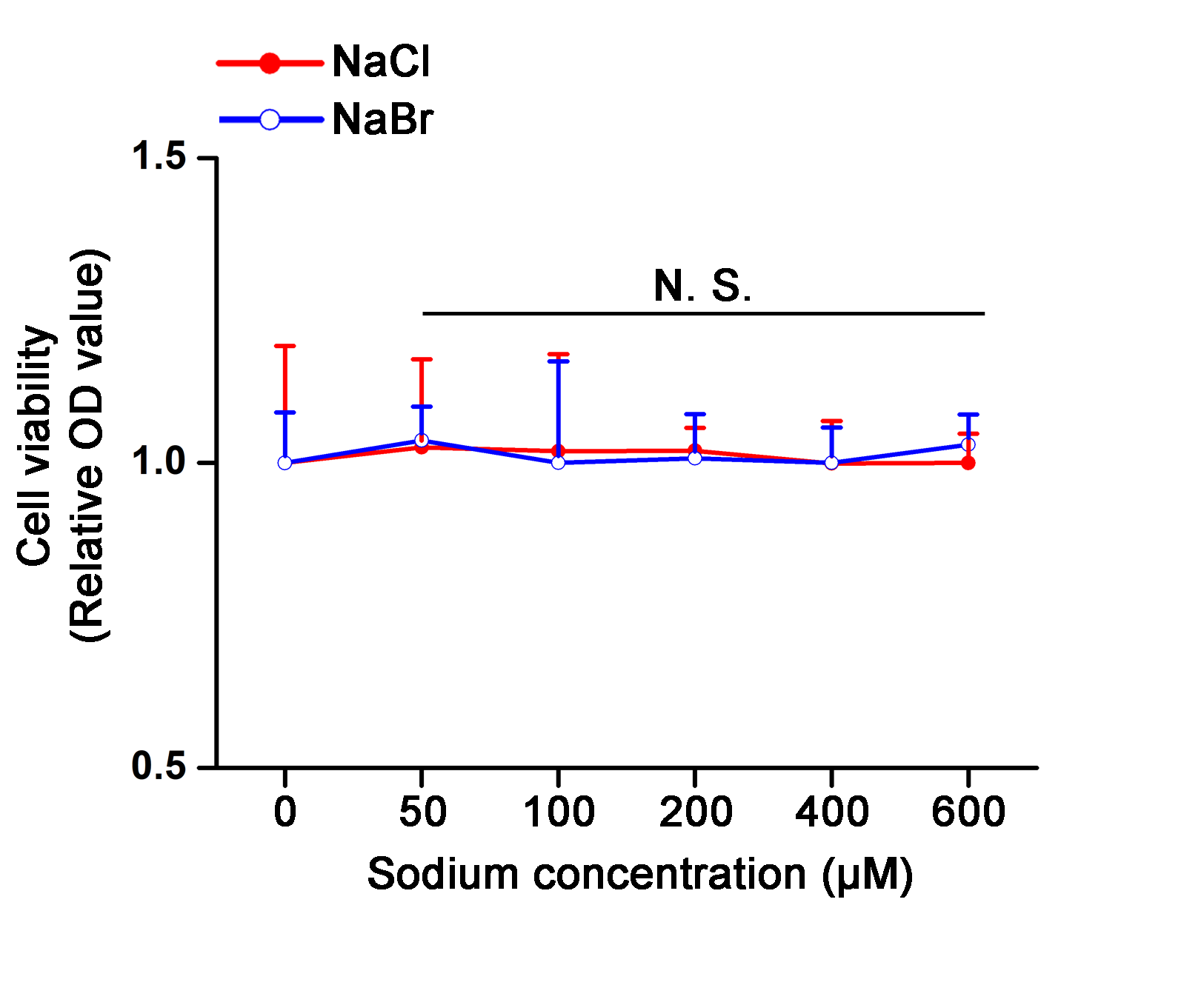

Supplement: Supplementary file 1 — Additional file 1: Figure S1. Bromide does not affect survival and apoptosis of rat neonatal primary cardiomyocytes. Rat neonatal primary cardiomyocytes were treated with NaBr at indicated doses for 24 h. (a) Cell viability was assessed by CCK-8 assay. [file 12860_2020_289_MOESM1_ESM.tif]

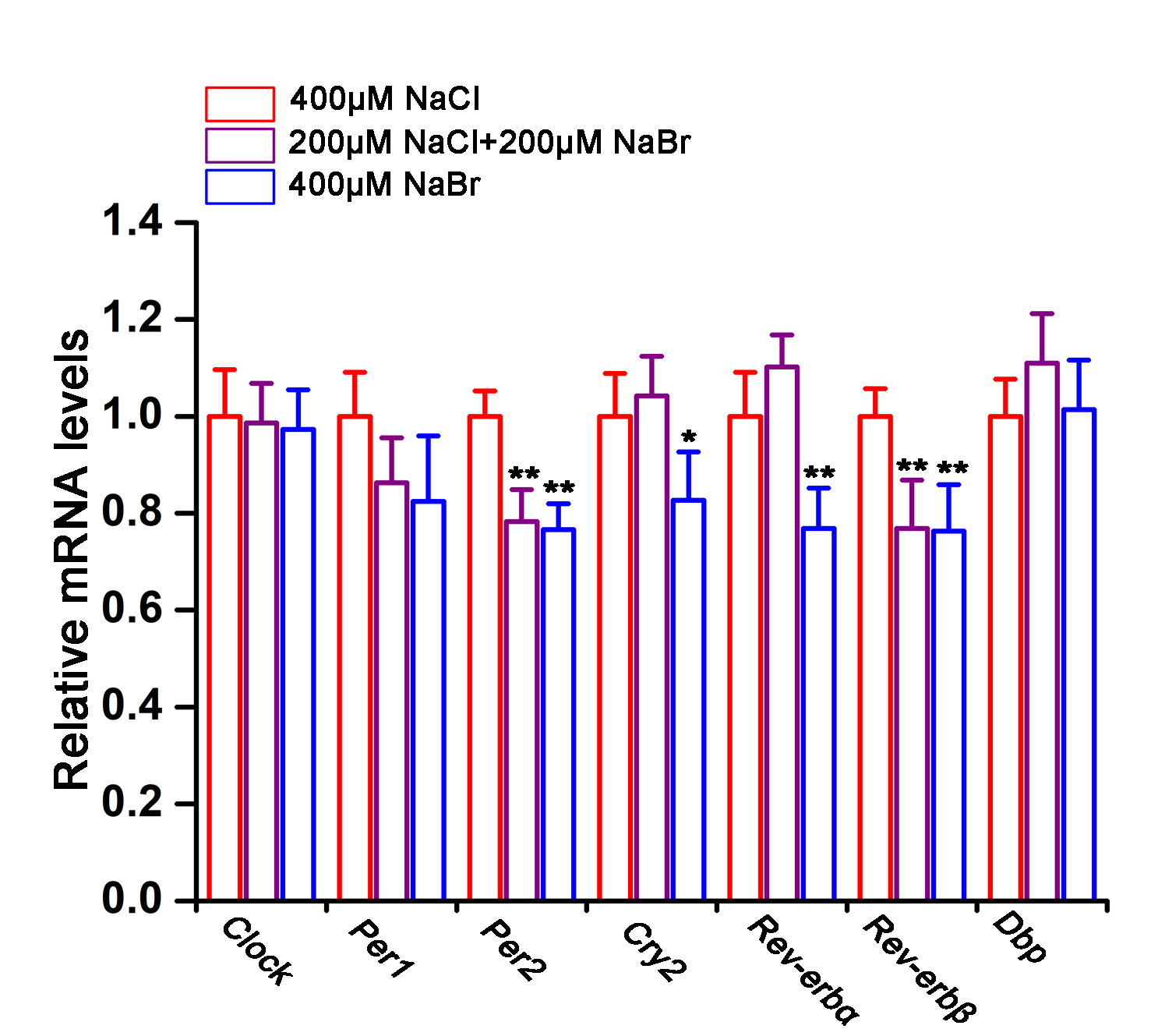

Supplement: Supplementary file 2 — Additional file 2: Figure S2. Bromide regulates clock gene expression in H9C2 cardiomyocytes. H9C2 cardiomyocytes were incubated with NaBr at indicated doses for 24 h. (a) RT-qPCR analysis of the mRNA expression levels of Clock, Per1, Per2, Cry1, Rev-erbα, Rev-erbβ and Dbp. *p < 0.05 and **p < 0.01 vs. NaCl group. n = 3. All the data were represented as the mean ± SD. [file 12860_2020_289_MOESM2_ESM.tif]

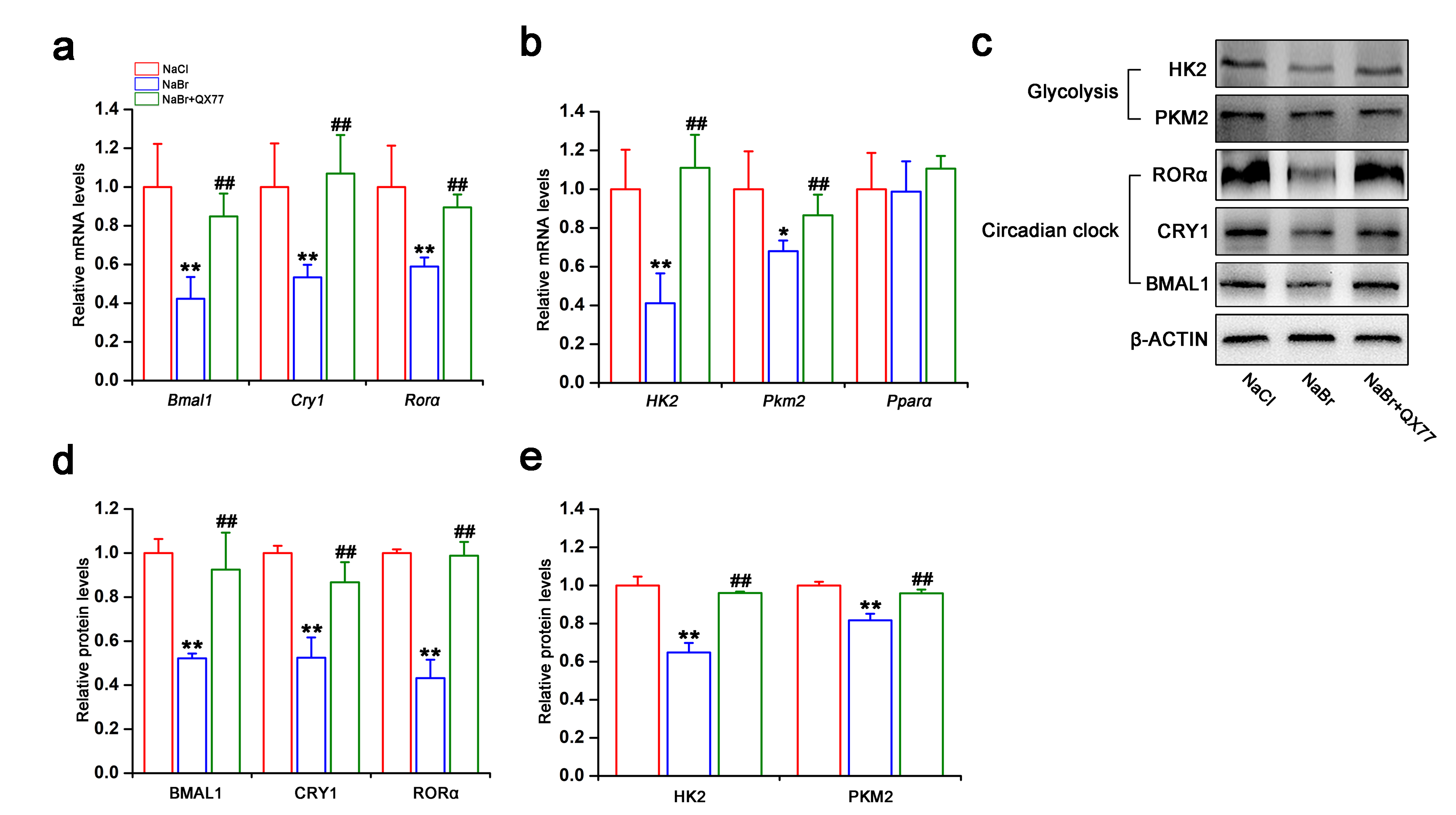

Supplement: Supplementary file 3 — Additional file 3: Figure S3. QX77 partially reversed the inhibitory effect of NaBr on the mRNA and protein expression levels of clock genes and glycolytic genes. H9C2 cardiomyocytes were treated with 400 μM NaBr in combination with or without 10 μM QX77 for 24 h. (a) RT-qPCR analysis of the mRNA expression levels of Bmal1, Cry1 and Rorα. (b) RT-qPCR analysis of mRNA expression levels of Hk2, Pkm2 and Pparα. (c) Western blot analysis of protein expression levels of BMAL1, CRY1, RORα, PKM2 and HK2. (d) Densitometric determinations of BMAL1, CRY1, RORα, (e) HK2 and PKM2. *p < 0.05 and **p < 0.01 vs. NaCl group, #p < 0.05 and ##p < 0.01 vs. NaBr group. n = 3. All the data were represented as the mean ± SD. [file 12860_2020_289_MOESM3_ESM.tif]

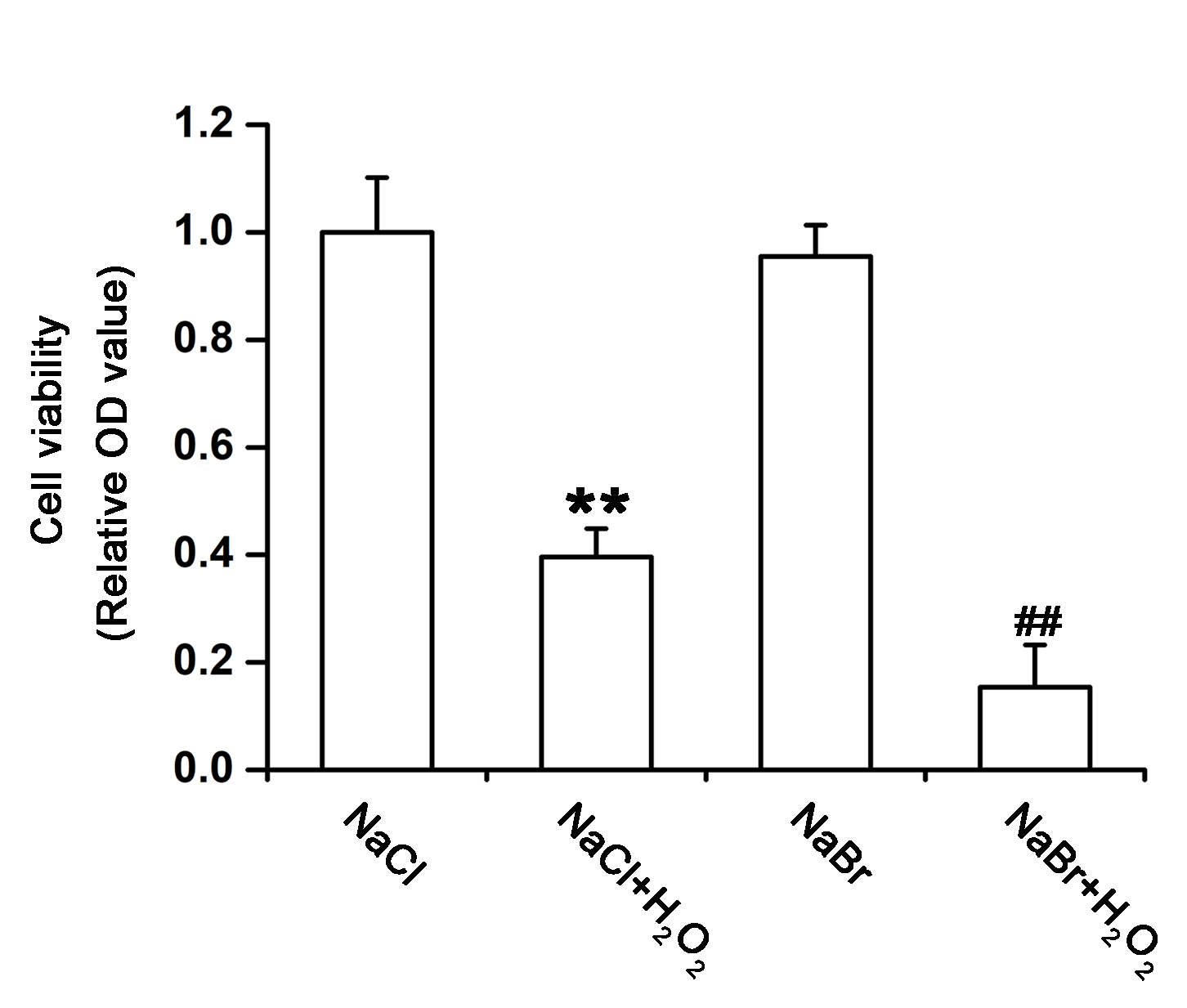

Supplement: Supplementary file 4 — Additional file 4: Figure S4. H9C2 cardiomyocytes were susceptible to H2O2 stimuli after treatment of NaBr. H9C2 cardiomyocytes were treated with NaBr with or without 1 μM H2O2 for 24 h. Cell viability was assessed by CCK-8 assay. **p < 0.01 vs. NaCl group, ##p < 0.01 vs. NaCl plus H2O2 group. n = 3. All the data were represented as the mean ± SD. [file 12860_2020_289_MOESM4_ESM.tif]

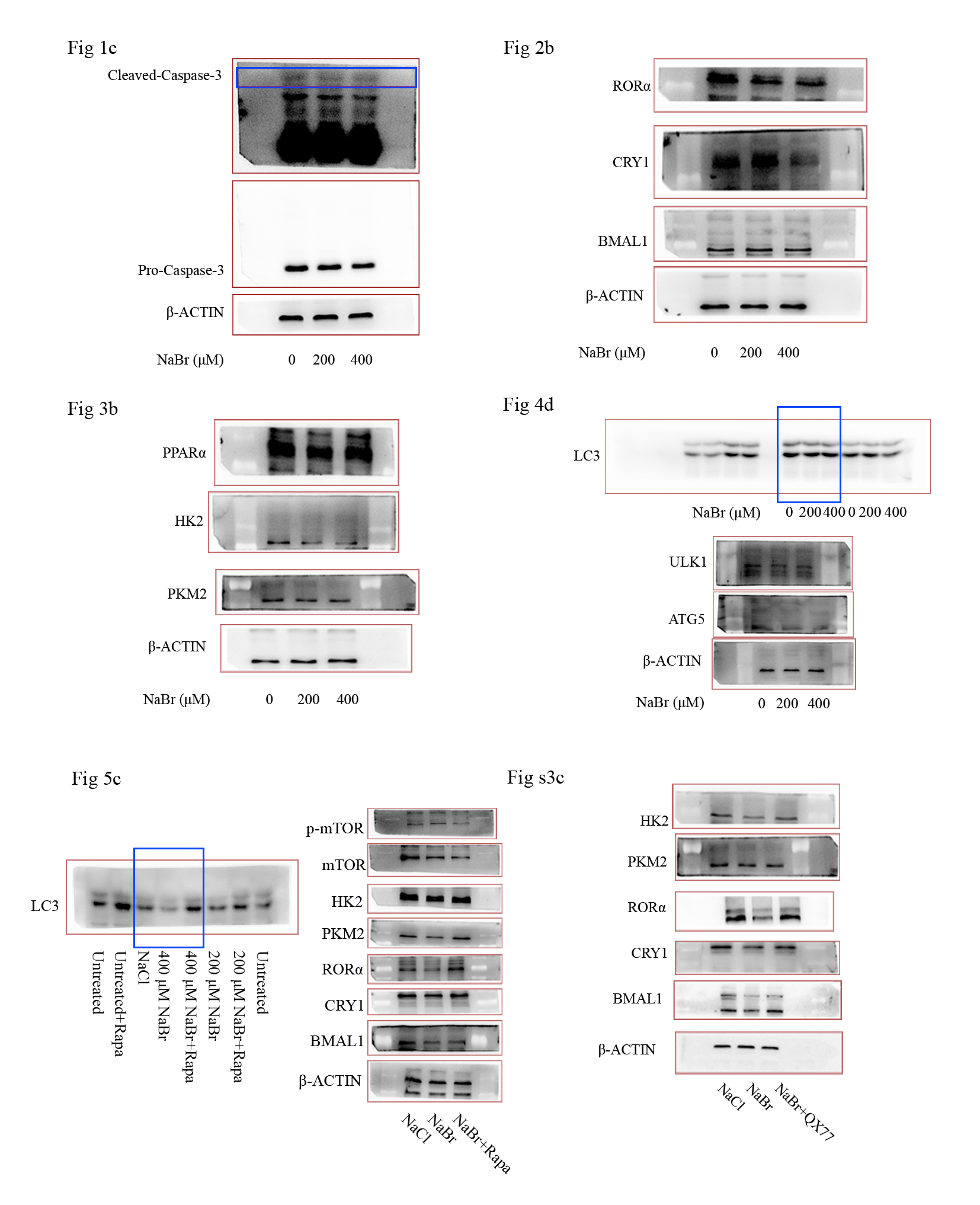

Supplement: Supplementary file 5 — Additional file 5: Figure S5. Uncropped images of the blots included in the Figures. [file 12860_2020_289_MOESM5_ESM.tif]
