# Supplementary material for: Bromide impairs the circadian clock and glycolytic homeostasis via disruption of autophagy in rat H9C2 cardiomyocytes
Source: BMC Mol Cell Biol. 2020 Jun 19;21:44. doi: 10.1186/s12860-020-00289-8 (PMC7304218; doi:10.1186/s12860-020-00289-8)
Supplement: Supplementary file 6 — Additional file 6: Table S1. Calculations of Amplitude and Phase shift in Fig. 2. Table S2. Calculations of Amplitude and Phase shift in Fig. 3. Table S3. Calculations of Amplitude and Phase shift in Fig. 4. Table S4. The list of primer sequences for qPCR analysis. [file 12860_2020_289_MOESM6_ESM.docx]

| Gene name | Amplitude | | *p* value for Amplitude | Phaseshift | | *p* value for Phaseshift | Δphase (NaBr vs. NaCl) | Δphase  Average |
| --- | --- | --- | --- | --- | --- | --- | --- | --- |
|  | NaCl | NaBr |  | NaCl | NaBr |  |  |  |
| *Bmal1* | 0.21 | 0.11 | 0.016164033 | -2.06 | -2.34 | 0.150573265 | -0.28 | -0.2233 |
|  | 0.16 | 0.14 |  | -1.96 | -2.09 |  | -0.14 |  |
|  | 0.19 | 0.11 |  | -1.77 | -2.03 |  | -0.26 |  |
| *Clock* | 0.05 | 0.15 | 0.003405324 | -1.78 | -13.36 | 0.000647174 | -11.59 | -10.096 |
|  | 0.07 | 0.13 |  | -1.60 | -11.73 |  | -10.13 |  |
|  | 0.09 | 0.15 |  | -1.21 | -9.78 |  | -8.58 |  |
| *Per1* | 0.23 | 0.27 | 0.088128325 | -0.58 | -0.26 | 0.014389232 | 0.32 | 0.2316 |
|  | 0.22 | 0.26 |  | -0.40 | -0.21 |  | 0.19 |  |
|  | 0.23 | 0.23 |  | -0.45 | -0.27 |  | 0.19 |  |
| *Per2* | 0.29 | 0.35 | 0.014460176 | -0.66 | -0.70 | 0.395545208 | -0.04 | 0.05287 |
|  | 0.31 | 0.37 |  | -0.65 | -0.60 |  | 0.05 |  |
|  | 0.33 | 0.38 |  | -0.65 | -0.51 |  | 0.14 |  |
| *Cry1* | 0.34 | 0.34 | 0.139537843 | 3.32 | 3.30 | 0.289981592 | -0.01 | 0.175 |
|  | 0.36 | 0.33 |  | 3.37 | 3.55 |  | 0.18 |  |
|  | 0.39 | 0.33 |  | 3.43 | 3.79 |  | 0.36 |  |
| *Cry2* | 0.32 | 0.33 | 0.568007384 | -3.37 | -3.67 | 0.240332949 | -0.30 | -0.426 |
|  | 0.30 | 0.28 |  | -2.96 | -3.45 |  | -0.49 |  |
|  | 0.28 | 0.24 |  | -2.53 | -3.02 |  | -0.49 |  |

**Table S1.** Calculations of Amplitude and Phase shift in Fig. 2.

| *Rev-erbα* | 1.07 | 1.03 | 0.546124065 | -5.67 | -5.07 | 2.83676E-05 | 0.60 | 0.57033 |
| --- | --- | --- | --- | --- | --- | --- | --- | --- |
|  | 1.14 | 1.10 |  | -5.63 | -5.06 |  | 0.57 |  |
|  | 1.21 | 1.17 |  | -5.58 | -5.04 |  | 0.54 |  |
| *Rev-erbβ* | 0.40 | 0.66 | 0.095649589 | -3.07 | -3.42 | 0.00041534 | -0.35 | -0.394 |
|  | 0.45 | 0.58 |  | -3.01 | -3.41 |  | -0.40 |  |
|  | 0.52 | 0.51 |  | -2.95 | -3.38 |  | -0.43 |  |
| *Rorα* | 0.18 | 0.14 | 0.025304057 | -1.43 | -1.99 | 0.080165343 | -0.56 | -0.3733 |
|  | 0.19 | 0.15 |  | -1.32 | -1.53 |  | -0.21 |  |
|  | 0.20 | 0.16 |  | -1.22 | -1.57 |  | -0.35 |  |
| *Dbp* | 0.79 | 0.86 | 6.39516E-05 | -3.34 | -3.69 | 0.007052032 | -0.35 | -0.3243 |
|  | 0.78 | 0.87 |  | -3.20 | -3.52 |  | -0.33 |  |
|  | 0.78 | 0.87 |  | -3.26 | -3.56 |  | -0.30 |  |

**Table S2.** Calculations of Amplitude and Phase shift in Fig. 3.

| Gene name | Amplitude | | *p* value for Amplitude | Phaseshift | | *p* value for Phaseshift | ΔPhase (NaBr *vs.* NaCl) | ΔPhase  Average |
| --- | --- | --- | --- | --- | --- | --- | --- | --- |
|  | NaCl | NaBr |  | NaCl | NaBr |  |  |  |
| *Hk2* | 0.29 | 0.46 | 0.437642813 | -3.45 | -5.99 | 0.000233689 | -2.54 | -2.3397 |
|  | 0.29 | 0.21 |  | -3.54 | -5.44 |  | -1.90 |  |
|  | 0.30 | 0.42 |  | -3.23 | -5.81 |  | -2.58 |  |
| *Pkm2* | 0.27 | 0.52 | 0.617244481 | -0.34 | -2.17 | 0.013337489 | -1.83 | -1.4984 |
|  | 0.45 | 0.25 |  | -0.79 | -1.92 |  | -1.12 |  |
|  | 0.26 | 0.36 |  | 0.15 | -1.40 |  | -1.54 |  |
| *Pparα* | 0.19 | 0.11 | 0.004450818 | 0.19 | 1.00 | 0.017072428 | 0.81 | 1.05276 |
|  | 0.16 | 0.12 |  | 0.17 | 0.93 |  | 0.76 |  |
|  | 0.19 | 0.13 |  | 0.17 | 1.76 |  | 1.59 |  |

**Table S3.** Calculations of Amplitude and Phase shift in Fig. 4.

| Gene name | Amplitude | | *p* value for Amplitude | Phaseshift | | *p* value for Phaseshift | Δphase (NaBr vs. NaCl) | Δphase  Average |
| --- | --- | --- | --- | --- | --- | --- | --- | --- |
|  | NaCl | NaBr |  | NaCl | NaBr |  |  |  |
| *Ulk1* | 0.19 | 0.24 | 0.03049082 | -4.35 | -5.48 | 0.007565867 | -1.13 | -1.0143 |
|  | 0.10 | 0.31 |  | -4.09 | -5.11 |  | -1.02 |  |
|  | 0.08 | 0.23 |  | -3.99 | -4.88 |  | -0.89 |  |
| *Gabarapl1* | 0.11 | 0.16 | 0.212811981 | -9.08 | -7.12 | 0.008738159 | 1.97 | 1.60033 |
|  | 0.12 | 0.19 |  | -8.29 | -7.08 |  | 1.21 |  |
|  | 0.13 | 0.12 |  | -8.19 | -6.56 |  | 1.63 |  |
| *Atg5* | 0.23 | 0.18 | 0.01075632 | 2.81 | 7.08 | 0.0011111 | 4.27 | 5.04133 |
|  | 0.24 | 0.17 |  | 3.31 | 8.51 |  | 5.20 |  |
|  | 0.24 | 0.13 |  | 3.35 | 9.00 |  | 5.65 |  |

**Table S4.** The list of primer sequences for qPCR analysis

| Gene | Primer sequence ( 5'-3' ) | |
| --- | --- | --- |
| *Bax* | Forward: | GAACTGGACAACAACATGGAG |
|  | Reverse： | CAAAGTAGAAAAGGGCAACC |
| *Bcl-2* | Forward: | AGGGGCTACGAGTGGGATA |
|  | Reverse： | CAGGCTGGAAGGAGAAGATG |
| *Caspase-3* | Forward: | GTATTGAGACAGACAGTGGAA |
|  | Reverse： | GTGCGGTAGAGTAAGCAT |
| *Bmal1* | Forward: | TGCCACCAACCCATACAC |
|  | Reverse： | TCTTCCCTCGGTCACATC |
| *Clock* | Forward: | GACAAGGACAAAGCAAAAAG |
|  | Reverse： | CAGGAAGCATAGACCCCA |
| *Per1* | Forward: | TTGCAAACGGGATGTGTTT |
|  | Reverse： | CAGGCGAGATGGTGTAGTAGAG |
| *Per2* | Forward: | TACACCACCCCTTACAAGCT |
|  | Reverse： | CACTGACACGGCAGAAAAAA |
| *Cry1* | Forward: | AAGCCCAACTGGACTGAG |
|  | Reverse： | AAAGGGGAGGGGAACTAT |
| *Cry2* | Forward: | TTGGGAAAGAACGGGATG |
|  | Reverse： | CGCTTGTAGGTAAGGGGTG |
| *Rev-erbα* | Forward: | GCTCCCCTTCTTCTGCTTC |
|  | Reverse： | CTGTCTTCCATGGCCACTT |
| *Rev-erbβ* | Forward: | AGAAGTGTCTGTCCGTGGG |
|  | Reverse： | AGGAGGGGTGCTTTTGAT |
| *Rorα* | Forward: | AAGAACCACCGAGAAGATG |
|  | Reverse： | GGAAAATGGAGTCGCACA |
| *Dbp* | Forward: | AGAGACGCAAGAAGACTCAAGG |
|  | Reverse： | ACAGCACGGTAGTGGGACAG |
| *Hk2* | Forward: | GGGACGACGGTACACTCAA |
|  | Reverse： | CCGCTAATCATCTTCTCAAACA |
| *Pkm2* | Forward: | GACACGCAATCCCCAGACA |
|  | Reverse： | CCAAGTTCACACGAAGATCAAC |
| *Pparα* | Forward: | AGTACAGATGAGTCCCCTGG |
|  | Reverse： | CGAATAGTTCGCCGAAAG |
| *Ulk1* | Forward: | GACAGCCTACAGGAGAAACCT |
|  | Reverse： | GGGGAGCCCACAGTAAATA |
| *Gabarapl1* | Forward: | ATCTGGATAAGAGGAAGTACC |
|  | Reverse： | GACAAAGAAGAATAAGGCG |
| *Atg5* | Forward: | GAGCCATACTATTTGCTTTTGC |
|  | Reverse： | TCAGGGGTGTGCCTTCAT |
| *Gapdh* | Forward: | TGGAAAGCTGTGGCGTGA |
|  | Reverse： | GGATACATTGGGGGTAGGAA |
